# Can the integration of sports and health industries drive the upgrade of the sports industry?—An empirical study based on Chinese-style modernization

**DOI:** 10.1371/journal.pone.0297974

**Published:** 2024-02-07

**Authors:** Jun Liu, Xun Lin, Fengwei Dong

**Affiliations:** 1 Institute of Modern Urban Public Administration, Shanghai University of Engineering Science, Shanghai, China; 2 ShuGuang Hospital, Shanghai University of Traditional Chinese Medicine, Shanghai, China; 3 RuiJin Hospital, Medical College of Shanghai Jiao Tong University, Shanghai, China; University of Naples Federico II: Universita degli Studi di Napoli Federico II, ITALY

## Abstract

**Background:**

In the course of China’s modernization, the sports industry’s advancement plays a dual role in enhancing national health and driving economic transformation. The integration and coalescence of the sports and health sectors have emerged as pivotal avenues for the structural elevation of China’s sports industry. Hence, empirically scrutinizing the influential mechanisms and outcomes of sports and health industry integration on the sports industry’s structural enhancement holds substantial practical significance.

**Method:**

This study formulates theoretical hypotheses regarding the impact mechanism of sports and health industry integration on the sports industry’s sophisticated industrial structure. Drawing insights from literature review and categorization, three dimensions—industrial integration, government role, and market mechanism—are delineated. Employing panel data spanning 2015 to 2020 from four primary Chinese cities, an econometric model is devised to empirically dissect the influence of sports and health industry integration on the sports industry’s structural advancement.

**Findings:**

The three pivotal explanatory variables—integration of sports and health industries, government role, and market mechanism—exert a positive influence on the sports industry’s sophisticated industrial structure. Notably, the impact of market mechanisms outweighs that of government roles, with government roles exhibiting comparatively weaker individual impact effects.

**Conclusion:**

The dynamic development process characterizing the structural advancement of China’s sports industry in first-tier cities exhibits positive and sustained developmental traits, with discernible convergence in trends. While market mechanisms demonstrate a more immediate and pronounced direct promotional effect than government roles, the enduring influence of government roles over an extended timeframe is evident from a dynamic long-term development perspective. Building upon these findings, the study suggests relevant stakeholders foster advanced sports industry structures by: refining the integration system of sports and health industries; fortifying the fundamental role of market mechanisms; and fully leveraging the government’s impact in promoting sports and health industry integration.

## 1. Introduction

Individual well-being is fundamental to societal civilization and progress and serves as a key indicator of the Chinese modernization paradigm. The Chinese government has instituted strategic measures to advance the modernization agenda, including fostering a Healthy China, prioritizing public health in development strategies, and expediting the establishment of a sports powerhouse. During China’s modernization journey, the enhancement and growth of the sports industry serve a dual purpose—advancing public health and facilitating economic transformation. As a burgeoning sector with dual social and economic advantages, it caters to evolving societal expectations for an improved quality of life, heightened physical well-being, and elevated health standards. Simultaneously, it significantly contributes to economic structural transformation, domestic demand expansion, employment promotion, and the cultivation of novel economic development impetus.

To achieve industrial structural modernization, China’s sports industry has implemented "sports +" and "health +" industrial integration, guided by the innovation, coordination, green, openness, and sharing development principles. In 2019, the "Opinions on Advancing National Fitness, Sports Consumption, and Facilitating the High-quality Development of the Sports Industry" emphasized the imperative to enhance the sports industry’s structure, diversify the array of sports health products, boost the share of the sports service sector, and propel the integrated development of sports and its associated industries. In 2020, the "Outline for Establishing a Sports Powerhouse Nation" advocated the adherence to the comprehensive health concept, enhancement of the entire sports industry chain, and facilitation of integrated development between sports and related sectors. The aforementioned policy documents demonstrate the Chinese government’s integration of the sports and health industries.

Industry integration and development constitute a crucial approach for advancing industrial structural enhancement. The amalgamation of sports and health industries proves to be an efficacious method in attaining the ’Healthy China’ objective, serving as a shared developmental goal for both sectors. It contributes to the establishment of a robust ecosystem for national fitness and health, concurrently fostering the advancement of China’s overarching health objectives. On one hand, the integration enhances physical fitness, prevents and manages chronic diseases, elevates quality of life and well-being, and delivers high-quality medical, healthcare, rehabilitation, and other product services, catering to a spectrum of health requirements. Conversely, the integration facilitates the dismantling of traditional industry barriers, fosters cooperation and exchange centered on public health requirements, nurtures novel health consumption patterns and market demands, drives the adoption of innovative health technologies and product development, establishes fresh industrial cluster advantages, and infuses new vigor into economic and social development.

Currently, guided by the Healthy China strategy, the ongoing integration of the sports and health industries entails continual adjustment of the structures of these two major sectors, fostering mutual integration and extension of industrial chains. This integration has emerged as a burgeoning force in creating and absorbing employment within nascent industries, novel forms, and innovative models within the secondary or tertiary sectors. The study examines whether the integrated development of sports and health industries can facilitate the enhancement of the sports industry’s structure. Additionally, it investigates the current status of the resultant impact.

## 2. Literature review

The exploration of industrial integration commenced in the 1970s, with Western developed nations taking the lead in the cross-integration development of the information industry, marking the inception of theoretical research on industrial integration [1]. By 2002, theoretical research on industrial integration had permeated Chinese academic circles, and in 2005, the concept of sports industry integration made its inaugural appearance in the research conducted by Chinese sports circles [2, 3].

Initially, industrial convergence employed three intersecting circles to depict the phenomenon of technological convergence across the "computer industry," "publishing and printing industry," and "broadcast and film industry" [1]. However, in recent years, various integration drivers have led to the proposition of theories such as technological innovation theory, market demand theory, market supply theory, and comprehensive factor theory [1]. The predominant view among Western scholars attributes the impetus for industrial integration to technological advancement and the easing of government regulation. As of now, a unified concept of industrial integration remains elusive. Starting from 2002, Chinese academic circles, exemplified by Li Wuwei, have actively engaged with and introduced the theory of industrial integration [1]. Some studies have explored the integrated development trend of the international information industry, financial industry, logistics industry, and energy industry, as well as brief theoretical analysis and some empirical evidence on the integrated development trend of China’s information industry, financial industry, and energy industry. Currently, research on the integration of the sports industry, both domestically and internationally, predominantly centers on four key aspects: the industrial connection of the sports industry, the developmental trajectory of integration, the practical integration with related industries, and the mechanism of integration [4].

(1) The industrial connection of the sports industry. The input-output relationship and its mode within the sports industry represent the structural correlation effects, discernible in the level, degree, and quality of the sports industry’s structural correlation [2]. Empirical studies have substantiated that the sports industry functions as a service industry, with its influence coefficient notably surpassing the induction coefficient. Its development necessitates input and support from various industries [3]. In the year 2020, researchers conducted an analysis of the coupling coordination relationship and driving factors between the sports industry and regional sustainable development in 11 provinces and cities in the eastern part of China from 2013 to 2017. The study revealed that the overall coupling coordination degree between the sports industry and the region in the eastern part of China was at a primary coordination level [4]. The spillover effects of coupling coordination in various regions were not pronounced, and regional innovation, industrial structure upgrading, and human capital were identified as potential drivers for enhancing the coordination between the two entities at the provincial and municipal levels. (2) The developmental trajectory of integration. Following the proposal of the concept of sports tourism abroad in 1996 [5], research on the integration of the sports industry commenced, entailing extensive deliberations on the connotation of sports integration, product classification, and its economic and social impacts. The industrial integration of sports represents a dynamic developmental process, and its collaboration with other industries embodies the essence of sports industrialization [6]. (3) The practical integration with related industries. Currently, the primary focus is on the integration of the sports industry with the tourism, cultural, electronics, media, and real estate industries. Notably, research on the integration with the tourism industry is the most developed. Given the inherent synergy between sports and tourism, their industrial integration emerges as a strategic approach to optimizing the benefits of the sports tourism industry [7]. Concerning the convergence of the digital and sports industries, pertinent research posits that the development of China’s online sports industry has entered a pivotal phase. This study conducts a multidimensional analysis, encompassing dimensions such as scale, structure, and employment, to scrutinize the current status of China’s online sports industry. Furthermore, a classification of activities within the online sports industry is undertaken. Some research advocates that China’s online sports industry must seize the business opportunities presented by the pandemic. This necessitates a transformation from an extensive model to a refined one, facilitated by policy support and the application of new technologies, with the objective of identifying and capitalizing on hotspots in online sports and health-related consumer behaviors [8]. (4) The mechanism of integration. The path mechanism for the integrated development of sports and related industries encompasses three evolution modes: the path of technology integration, the path of business integration, and the path of market integration [9]. Grounded in this path mechanism, there exist three reconstruction modes: extension and integration, infiltration and integration, and intra-industry restructuring and integration [10]. (5) The integration of sports and health sub -industries. The integrated development of the sports industry and the health service industry is substantiated and secured by policies, with the industrial integration mechanism evolving along three dimensions: business integration, technology integration, and market integration [11]. In contrast to the degree of integration between the sports industry and other sectors, the existing level of integration between the Chinese sports industry and the elderly care industry is notably low, standing at 0.6707, and ranking at the bottom [12]. Over the past five years, notable advancements have been achieved in empirical research on the integration impact of the sports industry on the pension, tourism, and cultural sectors. The studies have primarily centered on measuring the integration of the sports industry with related sectors, industry influence, and other relevant aspects [13, 14]. (6) The Impact of the COVID-19 Pandemic on the Convergence of the Sports and Health Industries. Existing research primarily analyzes the short-term pressures faced by the sports industry during the pandemic, focusing on secondary sub-sectors such as fitness and leisure, tourism, winter sports, as well as equipment manufacturing and sales. This research discusses the negative, temporally lagged effects of the pandemic on the convergence between these industries [15]. The study posits that due to the strong dependence of the sports industry on the natural and social environment, it is susceptible to the influence of unforeseen events such as infectious diseases. Consequently, the overall trend of convergent development is currently at a nadir during the pandemic. Nevertheless, it is asserted that this downturn will not alter the long-term goal of the convergence development between the two industries [15].

Building upon the aforementioned overview, recent years have witnessed a shifting focus towards scholarly exploration and advancement of the integration between the sports and health industries, aligning with the implementation of the Healthy China strategy. To date, existing research has not quantified the impact of the integration of the sports and health industries on the enhancement of the sports industry structure. Building on this gap in the literature, and anchored in the proposal of three theoretical hypotheses regarding the impact mechanism of the integration of sports and health industries on the enhancement of the sports industry structure, this study employs an econometric model to empirically test the impact of this integration on the upgrading of the sports industry structure.

## 3. Research hypothesis

This study aims to explore the effects of the integration of sports and health industries on the upgrading of the sports industry structure, and the influences of factors such as government role and market mechanisms on this process. Based on these research objectives, this study proposes hypotheses about how the integration of sports and health industries promotes advanced industrial structuring in the sports industry, and how government role and market mechanisms affect this outcome [16].

### 3.1. The promotion effect of the integration of sports and health industry on the upgrading of sports industry structure

Currently, existing studies posit an asymmetric symbiotic relationship between the sports and health industries. The interaction of these two major sectors culminates in a symbiotic organizational unit, where natural, social, and technological resources permeate, motivate, and transform one another. This mutual benefit relationship holds considerable significance. Research conducted by Wang Yan, Shen Keyin, and others suggests that the sports industry, in conjunction with other sectors, can dismantle technical barriers through system integration, resource amalgamation, technology infusion, and business format integration. This process promotes the optimization and integration of industrial resources, giving rise to a “sports + X” industry model. Such integration also facilitates the blending of adjacent business spaces, enriching the supply of “sports + X” consumer offerings. Hence, there exists a synergistic effect between the integration of the sports industry with other sectors and the alteration of the sports industry structure. Industry integration emerges as a pivotal variable in the structural upgrading of the sports industry. Building upon these perspectives, this study analyzes the impact mechanism of sports and health industry integration on the upgrading of the sports industry structure.

Firstly, the integration of the sports and health industries fosters collaboration and innovation between the two, effectively utilizing their natural, social, and technological resources. This synergy propels the enhancement of the industrial structure. The convergence of sports and health industries erases industry barriers, leading to the gradual blurring of industrial boundaries. Through the complementarity of superior industrial resources, knowledge improves synergy and efficiency, spurring the development of new technologies, products, and formats. The advent of these innovations alters the technical characteristics of original sports and health industry products and services, amplifying the productivity of various production factors within the sports industry. This, in turn, elevates enterprise production capacity and competitiveness, promoting overall economic effects and refining the structure of the sports industry.

Secondly, industrial integration empowers enterprises as market players to adeptly navigate dynamic market trends and evolving consumer demands. Enterprises in the sports and health industries, through this integration, can penetrate new markets and customer groups with heightened adaptability to emerging technologies and shifts in customer preferences. This expansionary approach contributes to increased sales and revenue, enabling further investment in the continual upgrading and modernization of industrial structures. It is evident that the integration of sports and health industries stimulates innovation within the sports industry, propelling progress and modernization of the industry’s structure, ultimately achieving advanced technological upgrading.

In conclusion, the integration of the sports and health industries forges a symbiotic relationship, creating a comprehensive ecological industrial system encompassing both sectors. In this process, the sports industry elevates its structure, enhancing its long-term sustainability. Thus, we propose the foundational hypothesis H1: the integration of sports and health industries plays a positive role in promoting the upgrading of the sports industry structure.

### 3.2. The effect of the role of the government and the market mechanism on the upgrading of the structure of the sports industry

Viewed through the lens of Chinese-style modernization, which is the adaptation of modernization based on China’s national context, a pivotal aspect of economic modernization lies in the establishment of an economic system that optimally harnesses both the market and government functions. According to Western economic perspectives, China’s current social and economic operations are realistically characterized by an incomplete amalgamation of government intervention and market behavior. This study, therefore, posits expanded hypotheses encompassing two distinct economic operation levels—government intervention and the market mechanism.

(1) The market, not being an omnipotent mechanism in the trajectory of market economy development, necessitates government macro-control in the face of market failures. The Chinese reform and opening experience underscore the visible hand of the government as crucial in ensuring economic development and industrial upgrades. The government’s influence permeates the market and related industries through policy directives, financial backing, and holistic coordination across diverse economic levels. Consequently, the sports industry, akin to other sectors, is significantly impacted by government intervention, manifesting through industrial policy regulations and direct/indirect investments. As the sports and health industries integrate into the national modernization discourse, government influence extends to industrial rules, regulations, and market access controls, profoundly shaping industry operation, market dynamics, and the investment and operational vitality of the sports industry. Simultaneously, government investments in novel sports industry products, services, and formats via financial support and incentives act as catalysts for the structural upgrading of the sports industry. In the integration of the sports and health industries, government intervention effectively guides industrial factor input matching and structural alignment, leveraging its macro-control role to propel sports industry development and structural enhancement.

Consequently, this study posits the second extended hypothesis H2: the role of government intervention fosters the enhancement of the sports industry structure, amplifying the impact of structural improvement resulting from the integration of the sports and health industries.

(2) In a market economy setting, the market mechanism assumes a decisive role in resource allocation within industries. Existing studies unanimously assert that any industry’s development hinges on market promotion, emphasizing the imperative role of market-oriented reforms in upgrading the sports industry structure. Within the process of sports industry modernization, the market primarily contributes to shaping industrial value, allocating industrial resources through competition for enhanced efficiency, optimizing industrial value by improving governance environments, and guiding industrial value through legal frameworks—a quartet of facets constituting good governance. The market mechanism plays a pivotal role in supplying sports industry products and services, primarily from market-oriented enterprises dominating the market. These enterprises play a supportive role in allocating public sports health resources. The market mechanism’s agility in responding to consumer needs, identifying profit opportunities, and fostering innovation propels the integration of the sports industry and health sector, culminating in the structural upgrading of the sports industry. Dynamic shifts in consumer health preferences and technological advancements in the sports and health industry create new demands and business opportunities, steering sports industry companies toward adjusting product or service structures, increasing corporate investments, and accelerating service operations’ upgrade. The vast Chinese health market provides enterprises with ample opportunities to unlock potential and stimulate growth by integrating innovative products from the sports and health sectors. The market mechanism ensures element mobility, competitive processes, and property rights incentives within the sports element and product markets, facilitating an optimal allocation of resource elements and ultimately propelling the structural upgrading of the sports industry. The collaborative development of sports enterprises collectively defines the industry’s trajectory, a path also influenced and adjusted by the market mechanism.

Therefore, the third expansive hypothesis H3 is advanced: the market mechanism propels the enhancement of the sports industry structure, amplifying the impact of structural improvement arising from the integration of the sports and health industries.

## 4. Research methods

### 4.1. Baseline model construction

This study primarily explores the impact of integrating urban sports and health industries on the sports industry structure’s advancement, initializing with the construction of a classic panel model.


$$SSJ_{it}=\alpha_{0}+\alpha_{1}D_{it}+\alpha_{2}GOV_{it}+\alpha_{3}MKT_{it}+\beta TSZB_{it}+\gamma_{it}$$ (1)

In the above formula (1), *SSJ*_*it*_ represents the level of advancement in the sports industry structure, *D*_*it*_ is the integration degree of the sports and health industries, *GOV*_*it*_ and *MKT*_*it*_ respectively signify the degree of government intervention and market mechanisms. *TSZB*_*it*_ functions as the control variable, representing residents’ consumption level and human resources. The coefficients *α* and *β* correspond to the core explanatory variables, while *γ*_*it*_ accounts for random error terms. This study designates the degree of integration between the sports and health industries, the government role (GOV), and market mechanisms (MKT) as the fundamental explanatory variables.

### 4.2. Variable selection

#### (1) Indicators for the upgrading level of the sports industry structure

The explanatory variable, the upgrading level of the sports industry structure, utilizes the industry-wide recognized advanced index for the sports industry structure. The progression of the sports industry structure from a low to high level signifies its dynamic developmental process. At its core lies the relationship between the enhancement of labor productivity and the proportion of relevant departments within the sports industry. Thus, this study assesses and analyzes the proportion and labor productivity ratio of the sports industry sector.


$$SSJ_{it}=\overset{n}{\underset{i=1}{\sum }}(\frac{X_{i}}{X})\left(\frac{LS_{it}-LS_{ib}}{LS_{if}-LS_{ib}}\right)$$

In the formula above, where *i* = 1, 2, 3…. *n* represents the number of institutions and units in the sports system, *X*_*i*_ is the operating income of institutions and units in the sports system, the *LS*_*it*_ is the labor productivity of institutions and units in the sports system, *LS*_*ib*_ is the labor productivity upon entering the initial stage of industrialization, and *X* is the total number of institutions and units in the sports system. Also, *LS*_*if*_ denotes the labor productivity at the completion of the industrialization stage. Based on the prevailing circumstance that most secondary industries in the sports industry are categorized under the tertiary industry, this study uses a per capita income of 854 US dollars as the starting point for the initial stage of industrialization and a per capita income of 8836 US dollars to mark the conclusion of the industrialization stage.

#### (2) Core explanatory variables

*1)Indicator of integration degree between sports industry and health industry*. Drawing from Ye’s model on sports industry integration [13, 14], we developed an evaluation index system encompassing sports and health industry integration and development. This system consists of eight dimensions, covering aspects like sports industry scale, contribution, support, and investment, along with health industry scale, demand, investment, and support, as detailed in Table 1.

**Table 1:** System indicators of sports industry and health industry.

Industrial system	Level 1 indicators	Specific dimensions	unit	Industrial system	Level 1 indicators	Specific dimensions	unit
Sports Industry System	economic contribution	Added value of sports industry	100 million yuan	Health industry system	economic contribution	health care spending	100 million yuan
		The added value of the sports industry as a percentage of the GDP of the year	%			total cost of health	100 million yuan
		Education, sports culture and entertainment expenditures	100 million yuan			Health and social work fixed asset investment growth	%
		Growth of Fixed Assets Investment in Culture, Sports and Entertainment Industry	%			Number of participants in basic medical insurance at the end of the year	million people
	Basic size	Listed companies in the sports industry	induvial		Basic size	Listed companies in the health industry	induvial
		Culture, sports and entertainment practitioners	million people			Health and social work practitioners	million people
		Sports industry registered enterprise	induvial			Medical institutions	induvial
		Sports social organization	induvial			Registered enterprises in the health industry	induvial

Utilizing the connotation and quality of data indicators, we calculated the integration degree between the sports industry and health industry based on their comprehensive evaluation index system. The integration model’s definition is presented in Formula (2), where U denotes the comprehensive score of the sports industry, and G represents that of the health industry. A lower fusion value implies weaker integration, while a higher value indicates better fusion. The fusion value falls within the range of (0,1).


$$\text{C}={\left[\frac{U\times G}{{\left(U+G\right)}^{2}}\right]}^{\frac{1}{2}}$$ (2)

The degree of industrial integration (D) assesses the virtuous circle and synergy between the sports and health industry systems. Formula (3) is employed for its calculation in this study.


$$\text{D}=\sqrt{C\times T}\;(\text{T}=\alpha \text{U}+\beta \text{G})$$ (3)

In Formula (3), T signifies the overall synergistic effect’s comprehensive score for both major industrial systems, with α + β equalling 1. The weights (α and β) for the sports and health industry systems are set to 0.5 each, reflecting their equal importance based on expert consultation.

*2)Indicators for government role and market mechanism variables*. The government role (GOV) and market mechanisms (MKT) represent the other two core explanatory variables under consideration. The government’s role is gauged by the proportion of city fiscal expenditure to the city’s GDP. In assessing market mechanisms, we adopt Fan Gang’s marketization index for pertinent Chinese cities due to its comprehensive measure of complexity.

Simultaneously, for enhanced model interpretation, urban consumption level (TSZB) is introduced as a control variable. The consumption level in the tertiary industry stands as a pivotal gauge for measuring industrial structure modernization. Elevated consumption levels are imperative to nurture diversified demand in sports and health consumption, thereby fostering the multi-factor integration of the sports and health industries. This necessitates reforms on the supply side of both industries, uplifting product supply quality and propelling the structural development of the sports industry towards sophistication and rationalization. In this study, the ratio of per capita consumption expenditure of residents to per capita regional gross domestic product serves as a representative measure of urban consumption levels.

### 4.3. Data source

To empirically analyze the impact of the integration of the sports industry and the health industry on the upgrading of the sports industry structure, this study leverages panel data spanning 2015–2020 from four first-tier cities in China—Beijing, Shanghai, Guangzhou, and Shenzhen. The primary data sources include the "Statistical Yearbook," "National Economic and Social Development Statistical Bulletin," "Sports Industry Development Bulletin," GuoTai Junan Securities Database, and the National Industrial and Commercial Enterprise Registration Database of each city. All variable data undergo logarithmic processing. The panel data encompasses six variables: the degree of integration between the sports industry and the health industry, the advanced structuring of the sports industry, consumer demand, market mechanisms, government role, and talent resources. Notably, five variables—degree of integration, advanced structuring, consumer demand, government role, and talent resources—are derived through specific calculations. The degree of integration and advanced industrial structuring follows prescribed formulas. Other indicators are computed based on corresponding formulas outlined in Table 2 (Consumer Demand = Per Capita Consumer Expenditure / Per Capita GDP, Government Role = Government Expenditure / GDP, Talent Resources = Number of Students in General Universities / Total Urban Population).

**Table 2:** Variable definitions and descriptive statistical analysis.

variable	Variable definitions	lnG and the variables +/-	average	standard deviation	maximum value	minimum value
lnG	Advanced structure of sports industry (Comprehensive index)		4.24549	0.88478	5.524006	2.83195
lnD	Integration degree of sports and health industry (Comprehensive index)	+	4.13619	0.25172	4.484427	3.50067
TSZB	Consumer demand (Per capita consumption expenditure of residents/ GDP per capita)	+	1.88405	0.41573	2.650033	1.50025
MKT	market mechanism (Marketization Index)	+	2.56158	0.13163	2.840247	2.36113
GOV	government role (Government Expenditure/GDP)	+	3.00253	0.15468	3.165164	2.66613
RC	human resources (Number of students in colleges and universities/total population)	+	1.60232	0.57888	2.58538	0.97427

## 5. Empirical analysis

### 5.1. Variable description analysis and basic model testing

To ensure the consistency of estimated results in each econometric model, this study conducted comprehensive data processing and econometric inspection before analysis. To mitigate multicollinearity issues among variables, the natural logarithm of two comprehensive indicators—namely, the advanced structure of the sports industry and the degree of integration of the sports and health industries—was employed. Simultaneously, for ease of constructing cross-multiplication items, both independent and control variables underwent centralization. Descriptive analysis and statistics were performed on the dependent variable, the advanced structure of the sports industry, and related variables, with specific results presented in Table 2.

Employing panel data, this study empirically analyzes the impact of integrating sports and health industries on the upgrading of the sports industry structure from both static and dynamic dimensions. For static dimensions, the Fixed Effect model was employed for testing due to the individual effects of each city in the panel data of four first-tier cities. Regarding dynamic dimensions, considering the path dependence in the upgrading of the sports industry structure and the relationship between the current and previous years, the System Generalized Method of Moments (SYS-GMM) was utilized for analysis and testing. In Model 2 (FE) and Model 5 (SYS-GMM), the regression coefficients of sports and health industry integration (lnD) are significantly positive at the 0.01 level, with statistical significance. This indicates that the enhancement of sports and health industry integration significantly influences the improvement of the sports industry structure, considering that the four first-tier cities represent the regions with the highest level of modernization in China. The secondary health industry continues to integrate deeply, with "Sports + Health" services and products becoming key participants in the urban health service market, optimizing the internal industrial structure layout of both the sports and health industries.

For the model’s robustness test, this study utilized the FGLS-CT model and IV-2SLS-RE model to test the regression results of Model 2 (FE). The VIF values for Models 1 through 5 in Table 3 are all less than 10, indicating no multicollinearity problem. The Modified Wald, Wooldridge, and Pesaran values of Model 3 are all significant at the 0.01 level, signifying inter-group heteroscedasticity, intra-group autocorrelation, and cross-sectional serial correlation. The FGLS-CT model was applied for regression analysis and testing under Models 3 and 4. The regression coefficient of sports and health industry integration (lnD) is significantly positive at the 0.01 level in both models, suggesting that sports and health industry integration plays a significant role in promoting the upgrading of the sports industry structure, aligning with the FE benchmark model’s regression results.

**Table 3:** Basic hypothesis model regression results.

Explanatory variables	POLS	Static FE	FGLS-CT	IV-2SLS-RE	SYS-GMM
	model 1	model 2	model 3	model 4	model 5
lnD	2.9582***	3.4087***	3.5910***	3.9292***	3.4073***
Integration of sports and health industry	(0.8190)	(0.8395)	(0.1830)	(0.7425)	(0.2007)
GOV	1.8824*	1.6951*	1.9765***	1.6259***	1.6012***
Government regulation	(1.2419)	(0.7382)	(0.2647)	(0.2236)	(0.3466)
MKT	9.5544***	8.1961***	8.6812***	9.8349***	13.1580***
Market mechanism	(3.2696)	(2.3292)	(0.3880)	(2.3729)	(1.6377)
RC	1.6966***	1.2921*	1.28491***	1.5098	1.1265
Human resource	(0.4770)	(0.5411)	(0.0398)	(0.6253)	(0.2038)
SZB	1.2788*	1.9108**	1.8896***	1.8897***	4.2370***
Consumer demand	(1.0957)	(0.8223)	(0.1595)	(0.4002)	(0.6308)
L, lnG	/	/	/	/	0.9026***
Lag term of dependent variable					(0.1663)
Cons	13.3359**	7.8696*	10.9382***	11.2305***	21.4658***
Constant term	(5.6213)	(3.6721)	(0.5449)	(2.2575)	(3.0926)
Hausman test	/	69.77**	152.67***	/	/
F/Wald	12.01***	16.53***	5828.21***	293.26***	110.32***
R^2^	0.8109	0.6596	/	0.9641	/

****p< 0.01,

**p< 0, 05,

* p< 0.1, standard error in brackets

Additionally, to test the model’s stability under dynamic conditions, SYS-GMM model tests AR(1) = 0.091, AR(2) = 0.09, and Sargen = 0.42, confirming the robustness of the model’s regression results. The combination of instrumental variables is effective, and there is no serial correlation of residuals. Under dynamic conditions, the regression coefficient of sports and health industry integration (lnD) remains significantly positive at the 0.01 level, indicating that sports and health industry integration continues to play a significant role in promoting the upgrading of the sports industry structure during the dynamic estimation model transformation. The R2 values of the related models ranged between 0.9641 and 0.6596, with significant F/Wald values, signifying good model fitting.

### 5.2. The impact of the integration of sports and health industries on the upgrading of the sports industry structure

As depicted in Table 3, the regression coefficient values for the degree of integration between the sports and health industries (lnD) span from 2.9582 to 3.9292. Across Model 1 to Model 4, a noteworthy and positive correlation at the 0.01 significance level is evident between the degree of integration (lnD) and the variable representing the advancement of the sports industrial structure (lnG). This suggests that a one-unit increase in the degree of integration of sports and health industries corresponds to an elevation of the sports industry structure advanced index by 2.9582 to 3.9292 units. Simultaneously, the regression coefficients of the integration degree (lnD) in the dynamic SYS-GMM model are all significantly positive and surpass the 0.1 level, signifying a substantial impact of sports and health industry integration on the sports industry in Chinese cities. While the impact of structural escalation varies in static and dynamic regression results, both exhibit positive and significant effects.

Concerning other control variables, the variables of consumption demand and human resources exert a discernible influence on promoting the upgrading of the sports industry structure. Consumer demand contributes to the structural enhancement of the sports industry to a certain degree, with positive and statistically significant coefficients in Model 1 to Model 5. This underscores that consumer demand strongly and positively propels the development of the sports industry structure towards a more advanced direction—a pivotal factor in industrial advancement. The coefficients of human resources display notable variations in statistical significance across different models and demonstrate different significance in static and dynamic regression results, indicating that the variable of human resources has a diverse impact on the upgrading of the sports industry structure. Notably, differences in the impact of the density of professional human resources in the sports and health industry in different cities on the industrial structure underscore its varying influence.

In conclusion, this study substantiates the fundamental hypothesis (Hypothesis 1) that the integration of the sports industry and the health industry can foster the upgrading of the sports industry structure. Furthermore, the explanatory variables in both the basic Model 1 to Model 3 and the extended Model 4 to Model 5—namely, the degree of integration of the sports industry and the health industry, government regulations, and market mechanisms—exhibit consistent positive or negative coefficients with significant characteristics. This alignment underscores the reliability of the model estimation results and ensures robustness. The L.lnG coefficient value in the dynamic SYS-GMM model 5, being less than 1, is significantly positive, indicating that the upgrading of the sports industry structure in China’s first-tier cities is in a dynamic development phase. While the integration of sports and health industries maintains a continuous positive promotional effect on sports industry upgrading, the impact effect displays a gradual narrowing trend. This sustained positive impact underscores the pivotal role of sports and health industry integration as a driving force for the advanced development of the sports industry.

### 5.3. The influence of the government’s role and market mechanism on the upgrading of the sports industry structure

Concerning the direct effects of the government’s role and the market mechanism, the two primary industrial development approaches consistently exhibit significance and positivity across Model 1 to Model 6. This reflects the robustness and reliability of the estimated results, attaining statistical significance and holding scientific significance. It is evident that the market mechanism exerts the most significant impact on the upgrading of the sports industry structure, whereas the government’s role contributes to the enhancement of the sports industry, albeit with a less conspicuous impact compared to the market mechanism. Model 6, as presented in Table 4, highlights a positive and significant correlation between the market mechanism and the advanced structure of the sports industry. This underscores that the market mechanism, as the primary means of adjusting industrial development, significantly promotes the development of the sports industry structure. Simultaneously, the positive and significant coefficients of the government’s role in each model indicate that the government in first-tier cities acts as a driving force in advancing the sports industry and plays a pivotal role in the development and evolution of the advanced sports industry structure. It is noteworthy that the static individual effect of the government is less significant than the dynamic effect. The static individual effect attains significance at the 0.1 level, while the dynamic effect achieves significance at the 0.01 level. This underscores that the impact of the government’s role on the upgrading of the sports industry structure is a medium-to-long-term dynamic development process, with a relatively weak impact at a single point in time.

**Table 4:** Regression results of synergy hypothetical model.

Explanatory variables	FGLS-CT	FGLS-CT
	model 6	model 7
lnD	14.4461***	23.0132***
Integration of sports and health industry	(1.9067)	(0.7379)
GOV	18.3926***	1.8879***
government role	(2.6649)	(0.0936)
MKT	1.8687**	38.7535***
market mechanism	(0.3870)	(0.6947)
lnD × GOV	4.6595***	/
Integration of sports and health industry × government role	(0.6549)	
lnD × MKT	/	9.2623***
Sports and health industry integration × market mechanism		(0.2467)
Wald	7442.31***	33919.95***

****p<0.01,

**p<0,05,

*p<0.1, standard error in brackets

Exploring the synergy between the government’s role and the market mechanism, specifically in the context of the sports and health industry’s integration with the government’s role, Model 6 demonstrates a positively significant regression estimate for the integration of the sports and health industry multiplied by the government’s role. This suggests that the visible hand of the government can positively influence and propel the integration and enhancement of the sports industry structure through specific regulations. Consequently, the research hypothesis 2 posited in this study gains empirical support, affirming that the government in China’s first-tier cities acts as a "booster," guiding the integration of sports and health industries and facilitating the upgrading of the sports industry structure. It underscores the government’s macro-control as a driving force, actively participating in the integration of sports and health industries, thereby contributing to the advancement of the sports industry. Regarding the synergistic effect between sports and health industry integration and the market mechanism, Model 7 unveils a positively significant regression coefficient for sports and health industry integration multiplied by the market mechanism. This coefficient surpasses the corresponding coefficient for sports and health industry integration multiplied by the government’s role in Model 6, indicating significance. It emphasizes that the market mechanism’s influence on promoting the advanced aspects of the sports industry is more pronounced, positioning the market mechanism as the primary force driving the structural upgrading of the sports industry. Thus, the research hypothesis 3 proposed in this study gains empirical support, affirming that in China’s first-tier cities, the market mechanism serves as a crucial catalyst for the advanced development of the sports industry structure, playing a leading role in its evolution.

Upon comparing the direct and synergistic effects of government roles and market mechanisms, it is evident that market mechanisms take a lead role in advancing the sophisticated structuring of the sports industry. Simultaneously, the government’s role assumes a guiding and augmenting function, providing crucial support in the integration of sports and health industries. These two components collaboratively propel the enhancement and evolution of the sports industry in first-tier cities. While market mechanisms exhibit a more pronounced and immediate promotional effect compared to government roles, emphasizing their crucial and direct impact, a dynamic, long-term perspective reveals that government roles exert a sustained influence over an extended duration.

## 6. Conclusion

The study’s findings underscore the dynamic nature of the sports industry structure upgrading in China’s first-tier cities amid the backdrop of Chinese-style modernization. This process exhibits positive and continuous developmental trends, displaying a certain degree of convergence in its momentum. The integration of the sports and health industries emerges as a proactive force positively impacting the advancement of the sports industry structure in these cities. Both government intervention and market mechanisms contribute to the upgrading of the sports industry structure, with market mechanisms playing a more pronounced role as the primary driving force. While the influence of government intervention on the sports industry structure’s advancement is weaker in individual effects compared to market mechanisms, it demonstrates relative stability in the medium to long-term dynamic process.

Drawing from the research outcomes, this study proposes that stakeholders focus on promoting the advanced structuring of the sports industry through the following three avenues:

1. Optimize and enhance the integration system of sports and health industries: Research affirms the positive impact of integrating sports and health industries on the advanced structuring of the sports industry [16, 17]. This supports enterprises in swiftly identifying related industrial elements and ensures the smooth entry of these elements into the corresponding industrial chain, facilitating the dynamic integration of the value chains of both major industries.
2. Reinforce the fundamental role of market mechanisms in advancing the advanced industrial structuring of the sports industry through the integration of sports and health industries: The study confirms that market mechanisms serve as the fundamental driving force for promoting the upgrading of the sports industry structure [18]. Future efforts should concentrate on expediting the establishment of an efficient, standardized, and fair competition-oriented national unified sports industry market [19]. This entails enhancing the efficiency of market resource allocation related to the sports and health industries and leveraging market mechanisms, entities, and capital effectively [20].
3. Maximize the influential role of the government in propelling the advanced structuring of the sports industry through integration with the health industry: The government must delineate its responsibilities and boundaries in driving the development of sports and health industries [21]. It should play a supportive role in service, regulation, and guidance, maintaining a balanced relationship with the market without overstepping its role or misplacing priorities [22, 23].

Acknowledging the study’s limitations, particularly in the imperfect business environment and internal industry structure of first-tier cities, future research endeavors should include these factors as control variables to enhance the empirical foundation for subsequent investigations.

In summary, against the backdrop of advancing Chinese-style modernization, this empirical analysis elucidates the impact of integrating the sports industry and health industry on the structural upgrading of the sports industry [24]. It contributes to a deeper understanding of the internal mechanisms driving the structural upgrading of China’s sports industry, particularly through the integration of sports and health industries, augmenting the empirical foundation within existing literature [25].

## Supporting information

S1 Data (XLSX)
